# Berberine Overcomes Gemcitabine-Associated Chemoresistance through Regulation of Rap1/PI3K-Akt Signaling in Pancreatic Ductal Adenocarcinoma

**DOI:** 10.3390/ph15101199

**Published:** 2022-09-28

**Authors:** Keisuke Okuno, Caiming Xu, Silvia Pascual-Sabater, Masanori Tokunaga, Haiyong Han, Cristina Fillat, Yusuke Kinugasa, Ajay Goel

**Affiliations:** 1 Department of Molecular Diagnostics and Experimental Therapeutics, Beckman Research Institute of City of Hope, Biomedical Research Center, Monrovia, CA 91016, USA; 2 Department of Gastrointestinal Surgery, Tokyo Medical and Dental University, Tokyo 113-8510, Japan; 3 Department of General Surgery, The First Affiliated Hospital of Dalian Medical University, Dalian 116004, China; 4 Institut d’Investigacions Biomèdiques August Pi i Sunyer (IDIBAPS), 08036 Barcelona, Spain; 5 Molecular Medicine Division, The Translational Genomics Research Institute, Phoenix, AZ 85004, USA; 6 City of Hope Comprehensive Cancer Center, Duarte, CA 91010, USA

**Keywords:** pancreatic ductal adenocarcinoma, Berberine, Gemcitabine, chemoresistance, Rap1/PI3K-Akt signaling pathway

## Abstract

Gemcitabine (Gem)-based chemotherapy is one of the first-line treatments for pancreatic ductal adenocarcinoma (PDAC). However, its clinical effect is limited due to development of chemoresistance. Various naturally occurring compounds, including Berberine (BBR), provide an anti-cancer efficacy with time-tested safety, individually and in combination with chemotherapeutic drugs. Accordingly, we hypothesized that BBR might enhance the chemosensitivity to Gem in PDAC. In this study, cell culture studies using MIA PaCa-2 and BxPC-3 cells, followed by analysis in patient-derived organoids were performed to evaluate the anti-cancer effects of BBR in PDAC. Considering that cancer is a significant manifestation of increased chronic inflammatory stress, systems biology approaches are prudent for the identification of molecular pathways and networks responsible for phytochemical-induced anti-cancer activity, we used these approaches for BBR-mediated chemosensitization to Gem. Firstly, Gem-resistant (Gem-R) PDAC cells were established, and the combination of BBR and Gem revealed superior anti-cancer efficacy in Gem-R cells. Furthermore, the combination treatment induced cell cycle arrest and apoptosis in Gem-R PDAC cells. Transcriptomic profiling investigated the Rap1 and PI3K-Akt signaling pathway as a key regulator of Gem-resistance and was a key mediator for BBR-mediated chemosensitization in PDAC cells. All cell culture-based findings were successfully validated in patient-derived organoids. In conclusion, we demonstrate that BBR-mediated reversal of chemoresistance to Gem manifests through Rap1/PI3K-Akt signaling in PDAC.

## 1. Introduction

Pancreatic cancer is one of the lethal malignancies and currently ranks as the seventh leading cause of cancer deaths worldwide, with 495,733 new cases and 466,003 deaths reported in 2021 worldwide [1]. The incidence and death rates for pancreatic cancer have also risen in the United States (US), with 60,430 new cases and 48,220 deaths in 2021 [2]. Although recent progresses in newer treatment regimens have somewhat improved the prognosis of patients with pancreatic cancer, the overall prognosis remains quite dismal, with 5-year overall survival (OS) rates as low as 8–10% [2,3,4]. Furthermore, pancreatic cancer is expected to become the second leading cause of cancer death in the US by 2030, and this trend is believed to continue even into 2040 when mortality is expected to continuously reduce for other cancers [5,6]. Since the initial randomized clinical trial in 1997, Gemcitabine (Gem) has been the key treatment regimen for patients with pancreatic ductal carcinoma (PDAC); however, the median OS with this single-arm treatment ranges from only ~6–7 months [7,8,9,10].

Accumulating evidence suggests that single agent treatments in such a fatal malignancy have been inadequate. Hence, a treatment regimen consisting of multiple drugs is likely going to be more effective as it will impact multiple pathways, will improve overall therapeutic efficacy, and is unlikely to result in the development of chemoresistance—the Achilles heel for most cancer drugs [11,12,13]. Accordingly, in order to improve the prognosis of patients with PDAC, several clinical trials have attempted to examine the efficacy of Gem-containing doublet regimens by using chemotherapeutic or targeted agents; however, most of these trials have yielded largely negative results [14,15,16,17]. The nab-paclitaxel doublet treatment has improved the OS vs. single-Gem regimen [10], as well as more recently, the triplet combination of 5-FU, irinotecan, and oxaliplatin (FOLFIRINOX) has shown improved survival outcomes in patients with PDAC [9]. However, the therapeutic benefits of these combination treatments are often compromised by the simultaneous drug toxicity, as well as added expense, which often limits their overall clinical efficacy [9,10,18,19,20,21,21,22]. Therefore, developing an optimal combination of safe and cost-effective therapeutic modalities is a major clinical challenge for PDAC treatment, which can facilitate improved survival outcomes in patients suffering from a lethal malignancy such as PDAC.

Since naturally occurring compounds provide an anti-cancer efficacy with time-tested safety, they are frequently used as an adjunctive supplementary therapy in various cancers, including PDAC [23,24,25,26,27,28,29,30,31,32,33]. Inflammation and stress affect cancer progression, and these compounds are also considered to be effective for inflammation and stress-induced disorders. During the last decade, numerous preclinical research studies have revealed that a combination of natural compounds and conventional chemotherapy not only enhances the anti-cancer therapeutic efficacy but also reduces the toxicity in PDAC [23,34]. In particular, several clinical trials and laboratory-based research have demonstrated such anti-cancer efficacy of various natural compounds including Escin, Boswellic acids, and Andrographis, when used in combination with Gem [35,36,37,38]. Similarly, our recent research has also revealed that Curcumin enhanced the chemosensitivity of PDAC cells to Gem by inhibiting PRC2-PVT1-c-Myc axis [39].

Berberine (BBR) is a natural plant alkaloid, which has been used traditionally to treat various conditions including bacterial diarrhea among the Chinese and native Americans [40,41]. Interestingly, studies in the past decade in laboratory experiments have highlighted a plethora of additional medicinal activities of BBR including its anti-cancer properties in various malignancies, including PDAC—all of which are orchestrated through multiple molecular mechanisms and pathways, such as cell cycle arrest, induction of apoptosis, caspase-independent cell death, and alterations in citrate metabolism [42,43,44,45,46,47,48]. Furthermore, more recent studies have also unveiled that BBR treatment also enhances chemosensitivity or reverses chemotherapeutic drug resistance in several cancers [49,50,51,52,53]. In view of this evidence, we hypothesized that BBR might enhance chemosensitivity to Gem through regulation of multiple oncogenic cellular signaling pathways to exert its anti-cancer activity in PDAC.

Herein, we performed systematic experiments using multiple Gem-resistant PDAC cell lines, followed by their study in patient-derived tumor organoids to evaluate the ability of BBR to enhance the chemosensitivity of Gem in PDAC. Furthermore, using systems biology approaches for cell line-based transcriptomic profiling datasets, we identified potential molecular mechanism(s) and key growth regulatory pathways that enhanced BBR’s anti-tumorigenic effects in attenuating the Gem-resistance in PDAC.

## 2. Results

### 2.1. Berberine Enhances the Chemosensitivity to Gemcitabine in Gemcitabine-Resistant PDAC Cells

First, to examine the combinatorial effects of BBR and Gem, the cell viability assays were performed using the parental cells with various concentrations of BBR, Gem, and their combination. The 50% inhibitory concentrations (IC_50_) of BBR were 16.5 μg/mL in MIA PaCa-2 and 19.0 μg/mL in BxPC-3, while IC_50_ of Gem were 588 nM in MIA PaCa-2 and 618 nM in BxPC-3 (Supplementary Figure S1). Next, to decide dose ratio of BBR and Gem in combination treatment, dose ratio of IC_25_, IC_50_, and IC_75_ of BBR vs. IC_50_ of Gem (BBR: Gem = 10: 600, 20: 600, 40: 600, respectively) was examined. In these treatments, the ratio of IC_75_ of BBR vs. IC_50_ of Gem (BBR: Gem = 40: 600) revealed the synergistic anti-cancer effect, with 0.82 and 0.45 combination index (CI) values, which were calculated by the Chou-Talalay equation at 50% inhibitory concentrations [54], in MIA PaCa-2 and BxPC-3. Because a CI < 1.0 value was conceived as a synergistic interaction, these data suggested a synergistic anti-cancer effect of BBR and Gem in PDAC cells. The IC_50_ of combined treatment with BBR and GEM were BBR: 12.1 μg/mL, Gem: 181 nM in MIA PaCa-2; and BBR: 9.2 μg/mL, Gem: 138 nM in BxPC-3 (Supplementary Figure S1), therefore, following experiments were performed at a concentration of BBR: 10 μg/mL, Gem: 150 nM, which is approximately the median IC_50_ of combination treatment in both PDAC cells.

**Figure 1:**
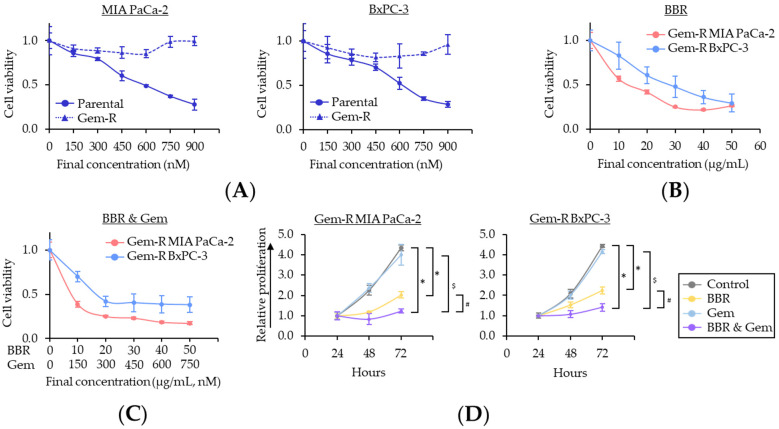
Berberine enhances the chemosensitivity to Gemcitabine-resistant PDAC cells in inhibiting cell proliferation. (**A**) Drug dose–response curves comparing cell viability following treatment with Gem in parental and Gem-R PDAC cells. Error bars are the mean ± SD; (**B**) Drug dose–response curves comparing cell viability following treatment with BBR in Gem-R PDAC cells. Error bars are the mean ± SD; (**C**) Drug dose–response curves comparing cell viability following treatment with combination of BBR and Gem in Gem-R PDAC cells. Error bars are the mean ± SD; (**D**) Cell proliferation assays in Gem-R PDAC cells with BBR, Gem, and their combination. Total viable cells were measured by CCK-8 assays on the indicated days. Error bars are the mean ± SD (* *p* < 0.05 vs. control, ^#^ *p* < 0.05 vs. BBR, ^$^ *p* < 0.05 vs. Gem). Gem-R, Gemcitabine-resistant; PDAC, pancreatic ductal adenocarcinoma; BBR, Berberine; Gem, Gemcitabine; CCK-8, Cell Counting Kit-8; SD, standard deviation.

To focus on BBR’s chemosensitization to Gem, Gemcitabine-resistant (Gem-R) PDAC cells were established as described in a previous study [39]. The cell viability assays using Gem-R PDAC cells revealed that no growth inhibition was observed in Gem-R PDAC cells up to 900 nM concentration range of Gem (Figure 1A). These data confirmed successful establishment of Gem-R PDAC cells (Gem-R MIA PaCa-2 and Gem-R BxPC-3) for subsequent experiments. Next, the anti-cancer activity of BBR was investigated in the growth inhibition of Gem-R PDAC cells, using Cell Counting Kit-8 (CCK-8) assays. BBR hampered the cell viability of Gem-R PDAC cells in a dose-dependent manner, and the IC_50_ of BBR in these cells were 14.6 μg/mL in Gem-R MIA PaCa-2 and 24.8 μg/mL in Gem-R BxPC-3, which were similar doses to each parental cell (Figure 1B). To further evaluate whether BBR enhanced chemosensitivity to Gem, the cell viability was examined in the combined treatment with BBR and Gem. The IC_50_ values of the combination treatment were BBR: 8.2 μg/mL, Gem: 123 nM in Gem-R MIA PaCa-2; and BBR: 17.1 μg/mL, Gem: 257 nM in Gem-R BxPC-3 (Figure 1C). In support of our hypothesis, for the proliferation assays with treatment of BBR and Gem individually, and their combination, the combination of BBR and Gem was significantly more effective in the inhibition of cell viability in Gem-R MIA PaCa-2 (fold change [FC] = 0.61 vs. BBR, *p* < 0.01; FC = 0.31 vs. Gem, *p* < 0.01) and Gem-R BxPC-3 (FC = 0.64 vs. BBR, *p* < 0.01; FC = 0.34 vs. Gem, *p* < 0.01; Figure 1D).

**Figure 2:**
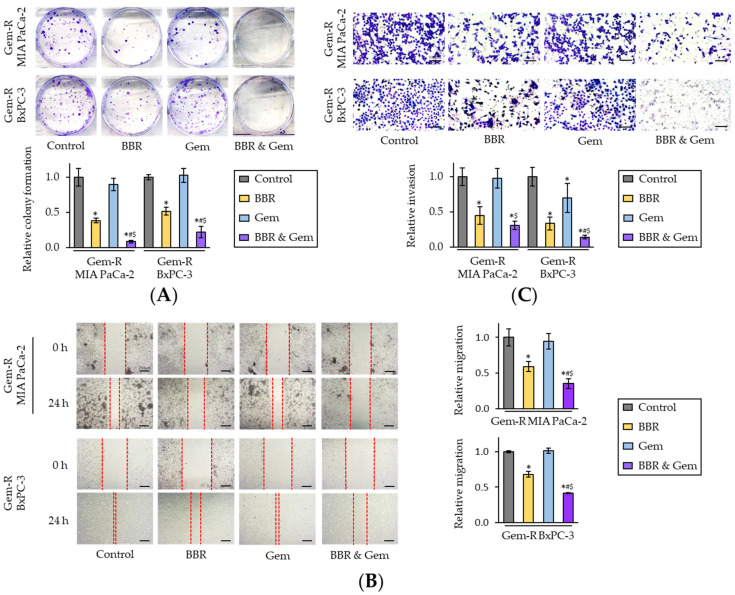
Berberine enhances the chemosensitivity of Gemcitabine-resistant PDAC cells by inhibiting colony formation, migration, and invasion. (**A**) Colony formation assays of Gem-R PDAC cells following treatment. The average (column) ± SD is indicated (* *p* < 0.05 vs. control, ^#^ *p* < 0.05 vs. BBR, ^$^ *p* < 0.05 vs. Gem); (**B**) Wound healing assays following in Gem-R PDAC cells. Images show representative areas (marked by red lines). Scale bar = 250 μm. The average (column) ± SD is indicated (* *p* < 0.05 vs. control, ^#^ *p* < 0.05 vs. BBR, ^$^ *p* < 0.05 vs. Gem); (**C**) Invasion assays following treatment in Gem-R PDAC cells. Scale bar = 100 μm. The number of cells was randomly counted at four fields, and then relative invasion ratios were calculated. The average (column) ± SD is indicated (* *p* < 0.05 vs. control, ^#^ *p* < 0.05 vs. BBR, ^$^ *p* < 0.05 vs. Gem). Images show representative fields on the membrane (magnification x100). Gem-R, Gemcitabine-resistant; PDAC, pancreatic ductal adenocarcinoma; BBR, Berberine; Gem, Gemcitabine; SD, standard deviation.

Next, the BBR’s ability to inhibit cellular clonogenicity, migration, or invasion was assessed—which are critical phenotypic features for cancer progression and metastases. The colony formation assays demonstrated that the combination treatment of BBR and Gem resulted in a significantly larger reduction of clonogenicity compared to either treatment individually in Gem-R MIA PaCa-2 (FC = 0.23 vs. BBR, *p* < 0.05; FC = 0.10 vs. Gem, *p* = 0.04) and Gem-R BxPC-3 cells (FC = 0.33 vs. BBR, *p* < 0.05; FC = 0.18 vs. Gem, *p* < 0.05; Figure 2A). Likewise, with regards to the wound healing and invasion assays, the combination of BBR and Gem significantly hampered cell migration (*p* < 0.05 vs. BBR; *p* = 0.01 vs. Gem in Gem-R MIA PaCa-2; *p* < 0.01 vs. BBR; *p* < 0.01 vs. Gem in Gem-R BxPC-3; Figure 2B) and invasion (*p* = 0.06 vs. BBR; *p* < 0.01 vs. Gem in Gem-R MIA PaCa-2; *p* = 0.01 vs. BBR; *p* < 0.01 vs. Gem in Gem-R BxPC-3; Figure 2C). Taken together, these data confirmed that BBR could significantly enhance the anti-cancer potential of Gem and even reverse Gem-resistance in PDAC cells.

### 2.2. Berberine and Gemcitabine Induce the G1 Phase Cell Cycle Arrest and Apoptosis in Gemcitabine-Resistant PDAC Cells

**Figure 3:**
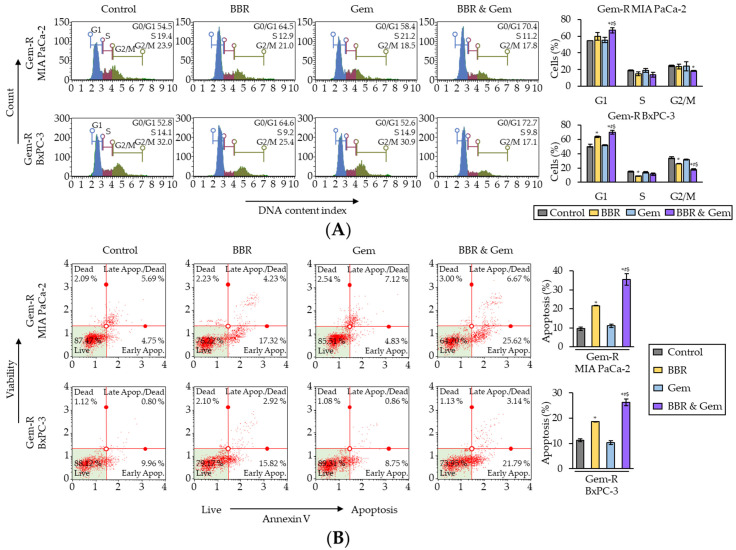
Berberine and Gemcitabine induce the G0/G1 phase cell cycle arrest and enhance cell apoptosis in Gemcitabine-resistant PDAC cells. (**A**) Representative images of cell cycle assays following treatment in Gem-R PDAC cells. The graph indicated the average ratio (column) ± SD of cells at each stage of cell cycle (* *p* < 0.05 vs. control, ^#^ *p* < 0.05 vs. BBR, ^$^ *p* < 0.05 vs. Gem). (**B**) Representative images of apoptotic cells that stained for annexin V assays following treatment in Gem-R PDAC cells. The average ratio (column) ± SD of cells undergoing apoptosis is indicated (* *p* < 0.05 vs. control, ^#^ *p* < 0.05 vs. BBR, ^$^ *p* < 0.05 vs. Gem). Gem-R, Gemcitabine-resistant; PDAC, pancreatic ductal adenocarcinoma; BBR, Berberine; Gem, Gemcitabine; SD, standard deviation.

To clarify the underlying mechanism for BBR-mediated reversal of Gem-resistance in PDAC cells, its impact on the cell cycle phase distribution was first evaluated by flow cytometry using propidium iodide as a probe. The cell cycle analyses showed that BBR increased the percentage of cells in G0/G1 phase in both Gem-R PDAC cells (*p* = 0.23 in Gem-R MIA PaCa-2; *p* = 0.04 in Gem-R BxPC-3; Figure 3A), whereas the combined treatment with BBR and Gem significantly induced the percentage of cells in G0/G1 phase in Gem-R MIA PaCa-2 (BBR vs. Combination, 59.9% vs. 67.0%, *p* < 0.05; Gem vs. Combination, 55.4% vs. 67.0%, *p* < 0.01) and Gem-R BxPC-3 (BBR vs. Combination, 63.7% vs. 70.1%, *p* < 0.05; Gem vs. Combination, 51.8% vs. 70.1%, *p* = 0.03; Figure 3A). Consistent with the results from the cell viability assays, BBR treatment significantly improved the anti-cancer potential by inducing cell cycle arrest in Gem-R PDAC cells. 

Next, Annexin V binding assays were performed to evaluate the impact of combined treatment with BBR and Gem on apoptotic rates. BBR significantly induced the cellular apoptosis compared to the untreated group in both Gem-R cells (*p* = 0.03 in Gem-R MIA PaCa-2; *p* = 0.03 in Gem-R BxPC-3; Figure 3B), while the combined treatment further enhanced the potential of apoptosis in both Gem-R MIA PaCa-2 (BBR vs. Combination, 21.6% vs. 35.5%, *p* < 0.05; Gem vs. Combination, 11.1% vs. 35.5%, *p* < 0.05) and Gem-R BxPC-3 (BBR vs. Combination, 18.7% vs. 26.3%, *p* < 0.05; Gem vs. Combination, 10.3% vs. 26.3%, *p* = 0.01; Figure 3B). Furthermore, when the expression of apoptosis-related genes was evaluated by quantitative reverse transcription polymerase chain reaction (qRT-PCR) the combined treatment significantly up-regulated the gene expression of Bax and Cyclin D1, and down-regulated Bcl-2 and Survivin (Supplementary Figure S2). Collectively, these data confirm that BBR reversed Gem-resistance through the cell cycle arrest and increased cellular apoptosis in Gem-R PDAC cells.

### 2.3. Rap1/PI3K-Akt Signaling Pathway Correlated with Gem-Resistance in PDAC Cells

**Figure 4:**
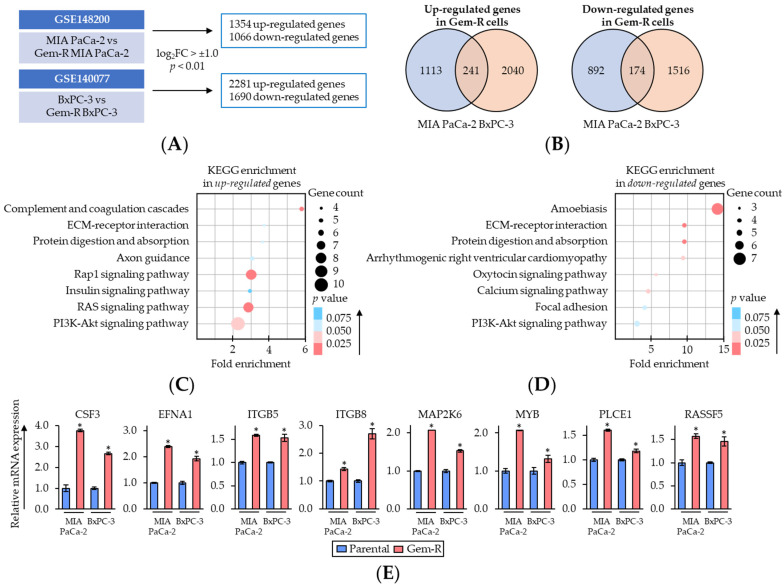
Rap1/PI3K-Akt signaling pathway correlates with Gemcitabine-resistance in PDAC cells. (**A**) Schematic of the differentially expressed gene discovery in Gem-R PDAC cells using GSE148200 and GSE140077. (**B**) Venn-diagram of up- and down-regulated expression (log_2_FC > ±1.0 and *p* < 0.01) of the genes in Gem-R PDAC cells. (**C**,**D**) Scatter plot of KEGG pathway enrichment analysis of up- (**C**) and down-regulated (**D**) genes in Gem-R PDAC cells. The number of differentially expressed genes in the pathway is indicated by the circle area, and the circle color represents the range of *p* value. (**E**) qRT-PCR analysis of key differentially expressed genes of Rap1/PI3K-Akt signaling pathway in parental and Gem-R PDAC cells. β-Actin mRNA expression was used as an internal control. The average (column) ± SD is indicated (* *p* < 0.05 vs. parental). Gem-R, Gemcitabine-resistant; PDAC, pancreatic ductal adenocarcinoma; BBR, Berberine; Gem, Gemcitabine; FC, fold change; SD, standard deviation.

To elucidate the key regulatory pathways associated with Gem-resistance in PDAC cells, the gene expression profiling results were compared between parental and Gem-R PDAC cells from transcriptomic profiling datasets (GSE148200 and GSE140077). This analysis revealed 1354 up- and 1066 down-regulated (log_2_FC > ±1.0, *p* < 0.01) genes in MIA PaCa-2, and 2281 up- and 1690 down-regulated genes in BxPC-3 (Figure 4A). Among them, there were 241 up-regulated and 174 down-regulated genes that overlapped in both PDAC cells (Figure 4B).

Next, Gene Ontology (GO) and Kyoto Encyclopedia of Genes and Genomes (KEGG) pathway enrichment analyses of these genes were performed using DAVID database (https://david.ncifcrf.gov/, accessed on 28 August 2022) [55,56] (Figure 4C,D). In the enrichment pathways, we focused on Rap1/PI3K-Akt signaling pathway as of the key pathways related to Gem-resistance in PDAC cells, because Rap1 and PI3K-Akt signaling interacted closely, and both of these pathways were significantly enriched among up-regulated genes (Rap1 signaling pathway, fold enrichment: 3.0, *p* = 0.02; PI3K-Akt signaling pathway, fold enrichment: 2.3, *p* = 0.03; Figure 4C). Thereafter, the expression of up-regulated genes included in the Rap1/PI3K-Akt signaling pathway was validated using qRT-PCR assays. The results of qRT-PCR assays revealed that the expressions of approximately 60% of genes (8/14 genes) were consistent with public transcriptomic profiling data (Figure 4E and Supplementary Figure S3). Collectively, these results suggested that Rap1/PI3K-Akt signaling pathway plays an important role in Gem-resistance in PDAC cells. Therefore, we hypothesized Rap1/PI3K-Akt signaling pathway as one of the key targets of the combined treatment with BBR and Gem and selected this pathway for subsequent experiments.

### 2.4. The Combined Treatment with Berberine and Gemcitabine Regulated the Activity of Rap1/PI3K-Akt Signaling Pathway in Gemcitabine-Resistant PDAC Cells

**Figure 5:**
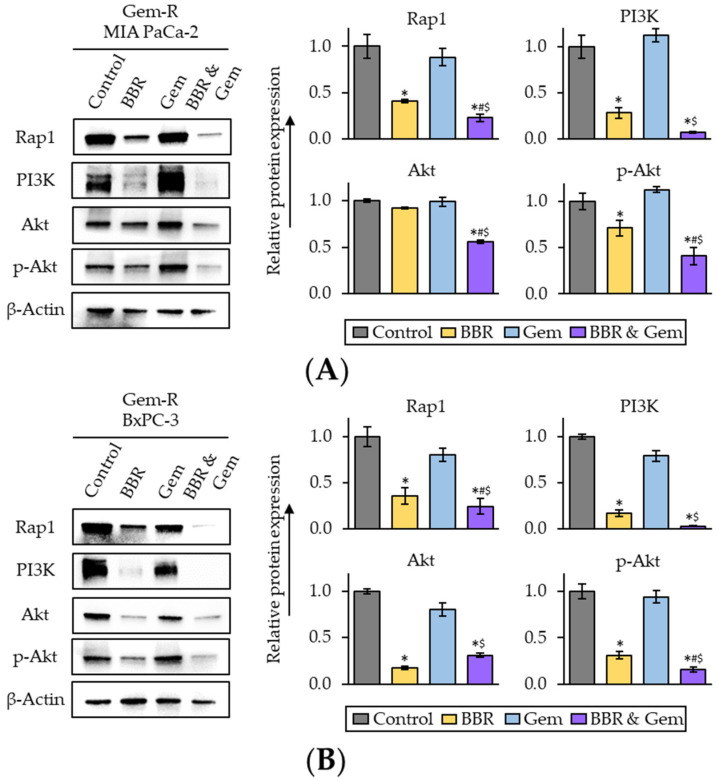
The combination treatment of Berberine and Gemcitabine regulates the activity of Rap1/PI3K-Akt signaling pathway in Gemcitabine-resistant PDAC cells. (**A**,**B**) Western immunoblotting of Rap1, PI3K, Akt, and phospho-Akt (ser473) (p-Akt) expression in Gem-R MIA PaCa-2 (**A**) and BxPC-3 (**B**) following treatment. The protein of β-Actin was used as an internal control. The average (column) ± SD is indicated (* *p* < 0.05 vs. control, ^#^ *p* < 0.05 vs. BBR, ^$^ *p* < 0.05 vs. Gem). Gem-R, Gemcitabine-resistant; PDAC, pancreatic ductal adenocarcinoma; BBR, Berberine; Gem, Gemcitabine; SD, standard deviation.

To examine whether Rap1/PI3K-Akt signaling pathway is a key target pathway of the combined treatment with BBR and Gem, we next performed Western blotting (WB) of key genes in the Rap1/PI3K-Akt signaling pathway following treatment with BBR, Gem, and their combination. The results of WB assays revealed Rap1 was significantly down-regulated by the combined treatment in both Gem-R MIA PaCa-2 (FC = 0.55 vs. BBR, *p* = 0.04; FC = 0.26 vs. Gem, *p* = 0.03) and Gem-R BxPC-3 (FC = 0.68 vs. BBR, *p* < 0.01; FC = 0.31 vs. Gem, *p* < 0.01; Figure 5A,B). Furthermore, PI3K expression was also significantly down-regulated by the combined treatment in both Gem-R MIA PaCa-2 (FC = 0.26 vs. BBR, *p* = 0.04; FC = 0.07 vs. Gem, *p* = 0.02) and Gem-R BxPC-3 (FC = 0.18 vs. BBR, *p* < 0.01; FC = 0.04 vs. Gem, *p* = 0.02; Figure 5A,B). For the protein expression level of Akt, we observed a significant down-regulation by the combined treatment in Gem-R BxPC-3; and this combined treatment attenuated phosphorylation of Akt at Ser473 (p-Akt) in both Gem-R MIA PaCa-2 (FC = 0.58 vs. BBR, *p* < 0.01; FC = 0.37 vs. Gem, *p* = 0.03) and Gem-R BxPC-3 (FC = 0.50 vs. BBR, *p* = 0.03; FC = 0.17 vs. Gem, *p* = 0.04; Figure 5A,B). Taken together, these results indicate that the Rap1/PI3K-Akt signaling pathway is potentially down-regulated by the combination treatment of BBR and Gem and potentially play a role in reversing Gem-resistance in Gem-R PDCA cells.

### 2.5. The Combination of Berberine and Gemcitabine Suppresses Spheroid Formation and Growth of Patient-Derived Organoids

**Figure 6:**
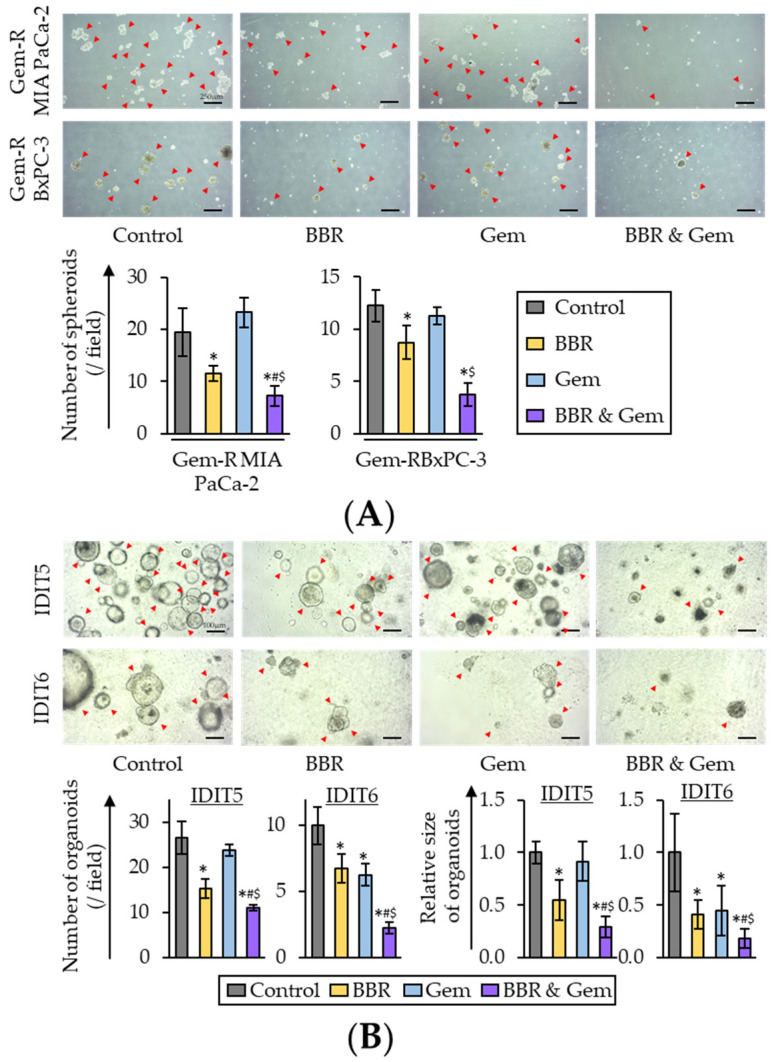
The combination of Berberine and Gemcitabine suppressed spheroid formation and growth of human PDAC tumor-derived organoids. (**A**) Representative images of sphere forming Gem-R PDAC cells following treatment. Scale bar = 250 μm (Magnification ×40). The average (column) ± SD is indicated (* *p* < 0.05 vs. control, ^#^ *p* < 0.05 vs. BBR, ^$^ *p* < 0.05 vs. Gem). (**B**) Representative images of tumor organoids following treatment. Scale bar = 100 μm (Magnification ×100). The average (column) ± SD is indicated (* *p* < 0.05 vs. control, ^#^ *p* < 0.05 vs. BBR, ^$^ *p* < 0.05 vs. Gem). Gem-R, Gemcitabine-resistant; PDAC, pancreatic ductal adenocarcinoma; BBR, Berberine; Gem, Gemcitabine; SD, standard deviation.

Three-dimensional (3D) cultures, such as 3D spheroids and tumor organoids, are conceived as physiologically superior to the conventional monolayer cell cultures for the study of anti-cancer agents [57,58]. Therefore, 3D culture models were finally used to validate our cell culture findings. First, the sphere formation assays were performed using Gem-R PDAC cells with treatment of BBR, Gem, and their combination. With regards to the sphere formation assays, the combined treatment with BBR and Gem significantly inhibited the sphere forming ability of both Gem-R MIA PaCa-2 (*p* = 0.04 vs. BBR; *p* < 0.01 vs. Gem) and Gem-R BxPC-3 (*p* = 0.10 vs. BBR; *p* = 0.02 vs. Gem; Figure 6A).

For further validation, tumor-derived organoid models from patients with PDAC were next utilized. PDAC patient-derived organoids (IDIT5 and 6) were generated as described in a previous study [59]. For these experiments, the viability of the tumor-derived organoids was evaluated following individual treatments with BBR and Gem, and their combination. The results revealed that BBR significantly inhibited the formation and growth of patient-derived organoids compared to untreated controls in both IDIT5 (number, *p* = 0.01; size, *p* < 0.01) and IDIT6 organoids (number, *p* = 0.03; size, *p* = 0.01; Figure 6B). Furthermore, the combined treatment with BBR and Gem significantly enhanced the anti-cancer activity in both the number of organoids (IDIT5, FC = 0.72 vs. BBR, *p* = 0.01; FC = 0.46 vs. Gem, *p* < 0.01; IDIT6, FC = 0.33 vs. BBR, *p* < 0.01; FC = 0.36 vs. Gem, *p* < 0.01; Figure 6B) and the size of organoids (IDIT5, FC = 0.53 vs. BBR, *p* = 0.03; FC = 0.32 vs. Gem, *p* < 0.01; FC = 0.44 vs. BBR, *p* < 0.01; FC = 0.41 vs. Gem, *p* = 0.01; Figure 6B). Collectively, these data highlighted that BBR significantly enhanced the anti-cancer potential of Gem, and yet again successfully validated our cell culture-based findings in these patient-derived tumor organoids.

## 3. Discussion

The current state of treatment for patients with PDAC involves combination chemotherapeutic regimens involving drug combinations such as FOLFIRINOX or gemcitabine with nab-paclitaxel, which are often accompanied by severe simultaneous drug toxicity and added expense [9,10,18,19,20,21,21,22]. Therefore, the development of optimal combination of safe and cost-effective therapeutic modalities is a major clinical challenge for treating patients with PDAC, which can help improve their survival outcomes. In this context, to overcome this clinical challenge, naturally occurring compounds provide an anti-cancer efficacy with time tested safety and cost effectiveness. In fact, more than 40% of anti-cancer drugs developed between 1981 and 2019 can be traced back in origin to naturally occurring compounds [60]. In addition, there are hundreds and thousands of studies published in the last two decades that show that the combination of chemotherapeutic drugs and such compounds can help enhance the anti-cancer therapeutic efficacy of conventional therapy, and at the same time reduce the simultaneous drug toxicity [27,61,62,63].

During the last few decades, a better understanding of the mechanisms responsible for Gem-resistance have led to the discovery of new therapeutic strategies for improving the survival and prognosis of patients with PDAC [64,65]. In this context, naturally occurring compounds have played a significant role and have been extensively studied for their chemosensitizing potential to various chemotherapeutic drugs [28,66,67]. In particular, BBR has recently gained increasing attention due to its anti-cancer potential by enhancing chemosensitivity and reversing chemotherapeutic drug resistance in cancer treatment. In breast cancer, BBR reversed chemoresistance through inhibition of the efflux function of ABC transporters [49] and autophagy through PTEN/Akt/mTOR signaling pathway [52]. Additionally, in glioblastoma, BBR reduced Temozolomide-resistance by inducing autophagy through ERK1/2 signaling [51]. For gastrointestinal cancers, BBR enhanced chemosensitivity to irinotecan via inhibition of NF-κB in colon cancer [53], and improved chemosensitivity to Cisplatin by enhancing cell apoptosis and repressing PI3K-AKt/mTOR signaling in gastric cancer [50]. Consistent with these previous reports, in this study, we found BBR-mediated reversal of chemoresistance to Gem on cell viability, clonogenicity, migration, and invasion in PDAC. Furthermore, in line with our findings, the enhanced anti-tumor effect of combined treatment with BBR and Gem was also confirmed in patient-derived tumor organoids. Overall, these findings indicate that BBR might be a promising anti-cancer compound for improving the sensitivity of Gemcitabine through multiple mechanisms. Our findings proposed the fundamentals of a new therapeutic modality for patients with PDAC, as not only adjunctive supplementary therapy of current chemotherapeutic regimens but also alternative therapy for patients who struggle to receive current combination chemotherapeutic therapies.

Rap1/PI3K-Akt signaling pathway plays a significant role in mediating the resistance of various chemotherapeutic drugs [68,69]. In particular, several previous studies have demonstrated that PI3K-Akt signaling pathway plays a pivotal role in Gem-resistance in various cancers [70,71,72]. Furthermore, recent studies revealed that PI3K-Akt signaling pathway is closely associated with tumorigenesis, tumor progression, and metastases in PDAC [73,74,75]. In line with these findings, in the present study, Rap1/PI3K-Akt signaling pathway was revealed as one of the key pathways related to Gem-resistance in PDAC cells. On the other hand, BBR’s properties on the regulation of PI3K-Akt signaling have been illustrated in other cancers [50,76,77]. Furthermore, interestingly, BBR also acted synergistically with PI3K inhibitor in colon cancer cells [78]. For the Rap1 signaling pathway, previously, it has been shown that BBR inhibits Rap1 signaling pathway which results in the suppression of platelet activation and thrombosis [79]. In line with these observations, the present study demonstrated that BBR induced cell cycle arrest and enhanced cellular apoptosis in Gem-R PDAC cells through the regulation of Rap1/PI3K-Akt signaling. More interestingly, we now report that the combined treatment with BBR and Gem enhanced this phenomenon and dysregulation of Rap1/PI3K-Akt pathway, which means BBR could improve the anti-cancer potential of Gem and even reverse Gem-resistance in PDAC cells.

The dose of BBR (10 μg/mL) in this study was similar to those in previous studies about the BBR’s chemosensitization in cancers [49,50,51,52,53]. In previous studies, 40–100 mg/kg oral dose of BBR was used in mice experiments [80,81]. When we translated it into a human equivalent dose using the Km factor [82], the practical dose for a 60 kg human might be 200–500 mg, which was an acceptable daily dose according to previous human clinical trials [83].

In this study, we investigated the BBR’s anti-tumor effect to enhance chemosensitivity to Gem and reverse Gem-resistance in PDAC cells, however, we would like to acknowledge some of the potential limitations of this study. First, although our RNA sequencing results identified many important molecular pathways, pathway analyses in this study included no data about PDAC cells treated with BBR and combination. The primary goal of this study was the first insight into the anti-tumor effects of BBR in PDAC, as well as its ability to enhance chemosensitivity to Gem in this malignancy. Therefore, in this study, we focused on Rap1/PI3K-Akt pathway, which has repeatedly been recognized as one of the key pathways associated with Gem-resistance in various cancers. Second, we were able to analyze organoids from only two patients. Taken together, future studies are warranted to investigate the further molecular mechanisms in terms of the BBR’s anti-tumorigenic effect to enhance chemosensitivity to Gem against PDAC cells, including the validation in the Gem-R tumor-derived organoids and animal models.

## 4. Materials and Methods

### 4.1. Cell Culture and Materials

Human PDAC cell lines, MIA PaCa-2 and BxPC-3, were obtained from the American Type Culture Collection (Manassas, VA, USA). The cell lines were cultured in RPMI medium (Gibco, Carlsbad, CA, USA) containing 10% fetal bovine serum (Gibco), and 1% penicillin and streptomycin (Gibco). Gemcitabine (Sigma-Aldrich, St Louis, MO, USA) was dissolved in dimethyl sulfoxide (DMSO), and then diluted to appropriate concentrations in the culture medium. Gemcitabine-resistant cell lines (Gem-R MIA PaCa-2 and Gem-R BxPC-3) were established by continuous culturing with increasing doses of Gem, as described in a previous study [39]. All cell lines were tested for mycoplasma contamination and authenticated using a panel of genetic and epigenetic markers for their genomic authenticity.

### 4.2. Herbal Preparations

Berberine used in the present study was Indian Barberry (Berberis aristate DC.) bark and root extract (DER 40:1, extraction solvent: methanol) standardized for the content of Berberine (97%), (EuroPharma, Green Bay, WI, USA). The herbal preparation quality of Berberine was authenticated using high-performance liquid chromatography in accordance with specifications. All analytical methods were validated for accuracy, selectivity, and precision. DMSO was used in the dilution of BBR to the stock concentrations, and before use, BBR was further diluted to appropriate concentrations in the culture medium.

### 4.3. Cell Counting Kit-8 Assays

For cell viability assays, 5 × 10^3^ cells were treated with various concentrations of BBR, Gem, and their combination for 48 h. Uniform DMSO concentrations were used in each treatment group. Following 48 h treatment, 10 μL of the CCK-8 solution (Dojindo, Kumamoto, Japan) was added and the cells were incubated for an additional 1 h, followed by measurement using a microplate reader (Tecan Trading AG, Männedorf, Switzerland). For cell proliferation assays, 3 × 10^3^ cells were grown for 24 h, and thereafter, cells were treated with various concentrations of BBR, Gem, and their combination. Their proliferation was evaluated at each time point using the CCK-8 assay kit.

### 4.4. Colony Formation, Wound Healing, and Invasion Assays

For the colony formation assays, cells (5 × 10^2^ cells/well) were seeded, followed by each treatment for 48 h. After culturing for 1 week in the complete medium, the number of colonies was counted following staining with 1% crystal violet and analyzing the colony counts by the Image-J 1.53q software (http://imagej.nih.gov/ij/index.html, accessed on 28 August 2022). For wound healing assays, after cells were grown to 80–90% confluency, wounds were created by scraping the cell monolayers, and subsequently treated with BBR, Gem, and their combination. Cells were observed under a microscope (magnification ×40). Twenty-four hours after scratching, the percentage wound closure was calculated using the Image-J 1.53q software. For the invasion assays, 3 × 10^4^ cells were grown in 24-well 8-μm pore size transwell chambers coated with Matrigel (BD Biosciences, Franklin Lakes, NJ, USA), followed by treatment. After 48 h, Diff-Quik staining was used to detect invading cells.

### 4.5. Cell Cycle and Apoptosis Assays

Cell cycle and apoptosis assays were performed using the Muse Cell Analyzer (EMD Millipore Corp, Hayward, CA, USA) according to the manufacturer’s instructions [25,26,33,84,85,86,87,88,89,90,91]. For the cell cycle assays, cells were seeded in 6-well plates and treated with BBR, Gem, and their combination for 48 h. Thereafter, cells were rinsed with ice-cold phosphate-buffered saline (PBS), fixed with 200 μL of ice-cold 70% ethanol and incubated at −20 °C for more than 3 h. Two hundred microliters of Muse^TM^ Cell Cycle Reagent (EMD Millipore Corp) were used in staining cells. The results were measured using the Muse Cell Analyzer. For the apoptosis assays, after 48 h treatment, 100 μL of Muse Annexin V & Dead Cell Reagent (Luminex Corp, Austin, TX, USA) were added into 100 μL of cell suspension by PBS. The apoptotic cell fraction was analyzed using the Muse Cell Analyzer.

### 4.6. Real-Time qRT-PCR and Primers

Total RNA extraction was performed using the Qiagen miRNeasy Kit (Qiagen, Hilden, Germany). The real-time qRT-PCR assays were performed using a SensiFAST SYBR Lo-ROX Kit (Bioline, London, UK) and the QuantStudio 6 Flex RT-PCR System (Applied Biosystems, Foster City, CA, USA). Using the β-actin gene as an internal control, the relative expression was quantified by the delta-delta Ct method [92]. The primers used in the present study are described in Supplementary Table S1.

### 4.7. Gene and Enrichment Pathway Discovery in Gemcitabine Resistant PDAC Cells 

To discover differentially expressed genes in Gem-R PDAC cells, systems biology approaches were performed by analyzing gene expression profiling differences between the parental and Gem-R PDAC cells, from two publicly available datasets (GSE148200 and GSE140077). These datasets were downloaded from the Gene Expression Omnibus database in their processed form (https://www.ncbi.nlm.nih.gov/geo/, accessed on 28 August 2022). A gene defined as differentially expressed if it had *p* < 0.01 and a Log_2_FC > ±1.0. Subsequently, enrichment analysis of GO and KEGG pathways was carried out using DAVID bioinformatic database (https://david.ncifcrf.gov/, accessed on 28 August 2022) [55,56]. A pathway was defined as enriched if the fold enrichment was more than 2.0, and scatter plots were generated based on enrichment pathway analysis using the DAVID database.

### 4.8. Protein Extraction and Western Blotting 

Total proteins were extracted from Gem-R PDAC cells using RIPA Lysis and Extraction Buffer (Thermo Fischer Scientific) with a protease inhibitor cocktail (Thermo Fischer Scientific). Proteins were separated by electrophoresis in a 10% Mini-PROTEAN TGXTM Precast Gel (BIO-RAD, Hercules, CA, USA), subsequently electro-transferred onto a 0.45 µm PVDF blotting membrane (Cytiva, Marlborough, MA, USA), followed by blocking in 5% bovine serum albumin (Sigma-Aldrich) solution for 1 h. Membranes were incubated overnight at 4 °C with diluted primary antibodies. Primary antibodies against Rap1 (1:1000, #2399; Cell Signaling Technology [CST], Danvers, MA, USA), PI3K (1:500, #4249; CST), Akt (1:1000, #4691; CST), and phospho-Akt (Ser473) (1:1000, #4060; CST) were used. The membranes were incubated with secondary antibodies (#7074 or #7076; CST) for 1 h. Immunoblots were visualized using an HRP-based chemiluminescence kit (Thermo Fisher Scientific) using Gel Imaging Systems (BIO-RAD). β-actin (1:1000, #58169; CST) was used as an internal control. The relative protein levels were evaluated using the Image-J 1.53q software.

### 4.9. Sphere Formation Assays

For sphere formation assays, 5 × 10^2^–1 × 10^3^ cells, following treatment for 48 h, were seeded in ultra-low attachment plates in serum-free DMEM-F12 medium (STEMCELL Technologies, Vancouver, BC, Canada) with B27 supplement (Gibco), basic fibroblast growth factor (bFGF; Gibco), and epidermal growth factor (EGF; STEMCELL Technologies). After 7–10 days, the spheroids were observed under a bright-field microscope (magnification ×40) and counted using the Image-J 1.53q software.

### 4.10. Patient-Derived Tumor Organoids

Tumor-derived organoids from PDAC patients were generated as described previously [59]. Following approval by the ethics committees of the institution, written informed consent was obtained from all patients. Patients were anonymously coded in accordance with ethical guidelines, as instructed by the Declaration of Helsinki. The tumor organoids were suspended in 40 μL of Matrigel (Corning, Tehama County, CA, USA) with 500 μL of PancreaCultTM Organoid Growth Medium (Catalog #100–0781, STEMCELL Technologies) containing EGF (STEMCELL Technologies) and prostaglandin E2 (STEMCELL Technologies) according to the manufacturer’s instructions. The organoids were treated with BBR (10 μg/mL), Gem (150 nM), and their combination (BBR: 10 μg/mL, Gem: 150 nM). After five days, the PDAC organoids that were more than 50 microns in diameter were observed under a microscope (magnification ×100). The number and size of organoids were measured using the Image-J 1.53q software.

### 4.11. Statistical Analysis

All statistical analyses were conducted using EZR version 1.55, which is a graphical user interface for R (R Foundation for Statistical Computing, Vienna, Austria, version 4.1.2) [93]. All experiments were performed as independent technical triplicates, and all data were expressed as mean ± standard deviation (SD). A two-sided Student’s *t*-test was used in the analysis of differences between continuous values of each group. *p* < 0.05 was considered statistically significant.

## 5. Conclusions

We firstly demonstrate BBR-mediated enhanced chemosensitization to Gem in PDAC cells using a systematic series of Gem-resistant cell culture and patient-derived tumor organoids experiments. We observed that Rap1/PI3K-Akt signaling pathway plays a critical role for Gem-resistance in PDAC, and BBR could overcome Gem-resistance via inhibition of key genes in Rap1/PI3K-Akt signaling pathway. Our findings could offer essential evidence of the combination treatment of BBR and Gem as a new safe and cost-effective therapeutic modality in this fatal malignancy.

## Data Availability

Data is contained within the article and supplementary materials.

## Supplementary Materials

The following supporting information can be downloaded at: https://www.mdpi.com/article/10.3390/ph15101199/s1, Supplementary Figure S1: Drug dose–response curves following treatment with BBR, Gem and their combination in parental PDAC cells; Supplementary Figure S2: qRT-PCR analysis of apoptosis-related genes in Gem-R PDAC cells following treatment with BBR, Gem, and their combination; Supplementary Figure S3: qRT-PCR analysis of differentially expressed genes of Rap1/PI3K-Akt signaling pathway in parental and Gem-resistant PDAC cells; Supplementary Table S1: Primer sequences and their PCR conditions in this study.
