# Supplementary material for: Berberine Overcomes Gemcitabine-Associated Chemoresistance through Regulation of Rap1/PI3K-Akt Signaling in Pancreatic Ductal Adenocarcinoma
Source: Pharmaceuticals (Basel). 2022 Sep 28;15(10):1199. doi: 10.3390/ph15101199 (PMC9611392; doi:10.3390/ph15101199)
Supplement: Supplementary file 1 [file pharmaceuticals-15-01199-s001.zip › pharmaceuticals-1903720-supplementary.pdf]

SUPPLEMENTARY FIGURES

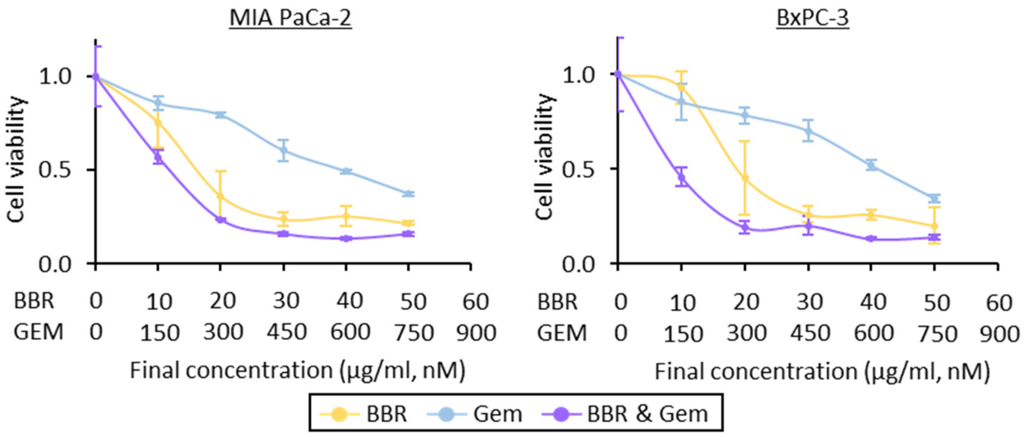

Supplementary Figure S1: Drug dose response curves determined by CCK-8 assays comparing cell viability following treatment with BBR, Gem and their combination for 48 hours in parental PDAC cells. Error bars are the mean ± SD. PDAC, pancreatic ductal adenocarcinoma; BBR, Berberine; Gem, Gemcitabine; CCK-8, Cell Counting Kit-8; SD, standard deviation.

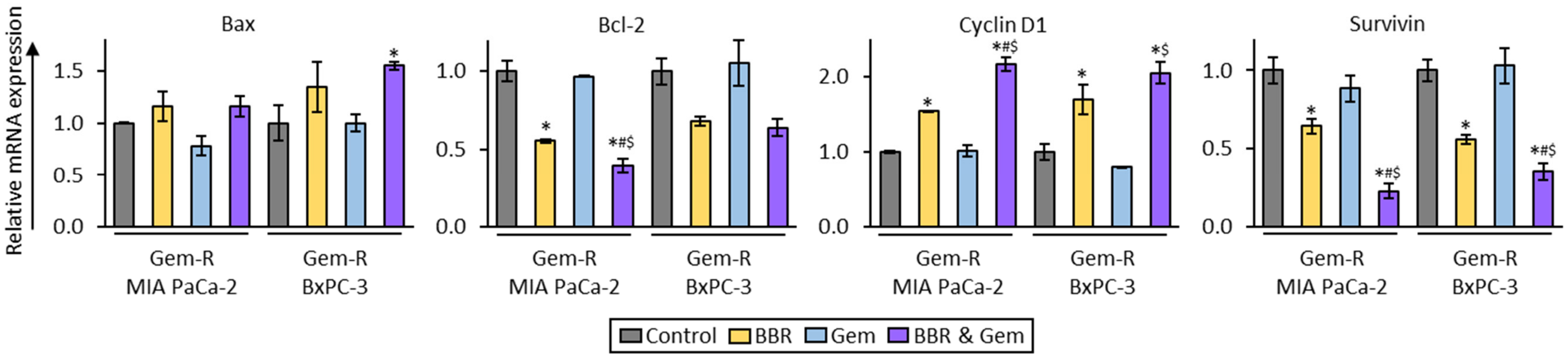

Supplementary Figure S2: qRT-PCR analysis of apoptosis-related genes (Bax, Bcl-2, Cyclin D1, and Survivin) in Gem-R PDAC cells following treatment with BBR, Gem, and their combination for 48 hours. Relative expression was calculated using β-Actin mRNA expression as an internal control. The average (column) ± SD is indicated (*$P < 0.05$ vs control, $^{\#}P < 0.05$ vs BBR, $^{\$}P < 0.05$ vs Gem). Gem-R, gemcitabine resistant; PDAC, pancreatic ductal adenocarcinoma; SD, standard deviation.

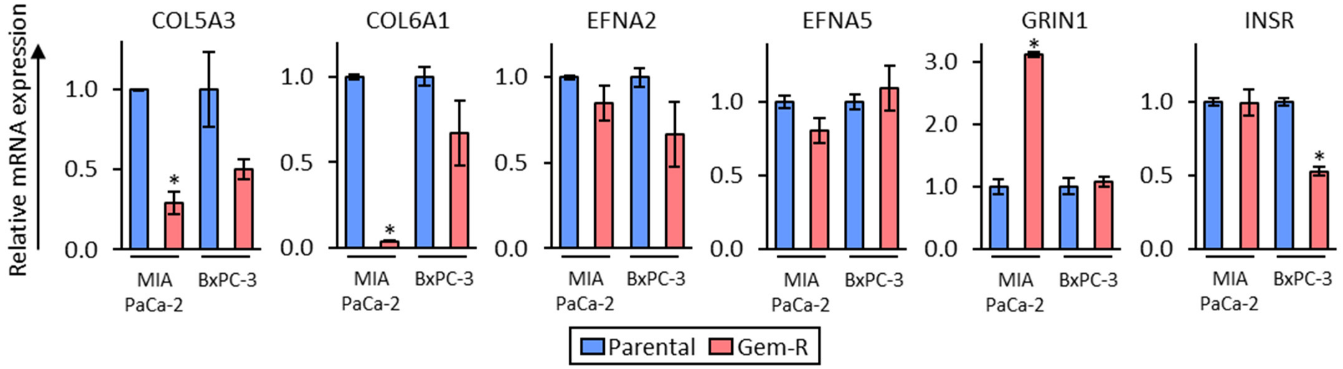

Supplementary Figure S3: qRT-PCR analysis of differentially expressed genes of Rap1/PI3K-Akt signaling pathway in parental and Gem-resistant PDAC cells. Relative expression was calculated using β-Actin mRNA expression as an internal control. The average (column) ± SD is indicated (**P* < 0.05). Gem-R, gemcitabine resistant; PDAC, pancreatic ductal adenocarcinoma; SD, standard deviation.

Supplementary Table S1: Primer sequences and their PCR conditions in this study

Gene	Sense	Antisense	Size of the PCR products (bp)	Annealing temp (°C)	GenBank accession No
COL5A3	GTGGCCGTCAGCATAGATGG	TGAATGTCTCCCTCGAAAGTCTT	158	60	NM_015719
COL6A1	ACACCGACTGCGCTATCAAG	CGGTCACCACAATCAGGTACTT	90	60	NM_001848
CSF3	GCTGCTTGAGCCAACTCCATA	GAACGCGGTACGACACCTC	285	60	NM_000759
EFNA1	TCAGGCCCATGACAATCCAC	GTGACCGATGCTATGTAGAACC	79	60	NM_004428
EFNA2	CGGTGGAGGTGAGCATCAAT	AGCGGCGCCCCATAGT	61	60	NM_001405
EFNA5	CTACATGGTGAACTTTGATGGCT	GAGGCCGGTTACATTCCCA	80	60	NM_001962
GRIN1	ACGCCATCCTAGTTAGCCATC	GCACGGGTATGCGGTAGAAG	93	60	NM_021569
INSR	AAGTGCATCCCTGAGTGTCC	ATTGTTGCCTCCTCGAATGT	201	60	NM_000208
ITGB5	GGAAGTTCGGAAACAGAGGGT	CTTTCGCCAGCCAATCTTCTC	106	60	NM_002213
ITGB8	GTGAAAGTCATATCGGATGGCG	GCTATCAAGAGCGAGATGAGACG	86	60	NM_002214
MAP2K6	AAACGGCTACTGATGGATTTGG	CAGTGCGCCATAAAAGGTGAC	78	60	NM_002758
MYB	ATCTCCCGAATCGAACAGATGT	TGCTTGGCAATAACAGACCAAC	157	60	NM_005375
PLCE1	CAACGCTGTCATGGAGTTCTT	TGGTCTCAATATCAGACTGGTCC	89	60	NM_016341
RASSF5	GGGCATGAAACTGAGTGAAGA	TGGCATCATAGATGGACTGGG	116	60	NM_182665
Bax	CCAGCTCTGAGCAGATCATG	TCAGCCCATCTTCTTCCAGA	392	60	NM_138763
Bcl-2	GGTGCCACCTGTGGTCCACCTG	CTTCACTTGTGGCCCAGATAGG	459	60	NM_000633
Cyclin D1	ACCTGGATGCTGGAGGTCT	GCTCCATTTGCAGCAGCTC	241	60	NM_053056
Survivin	TGCCTGGCAGCCCTTTC	ATGAAGCCAGCCTCGGC	108	60	NM_001168
β-actin	AGAGCTACGAGCTGCCTGAC	AGCACTGTGTTGGCGTACAG	184	60	NM_001101
